# Impact of Clinicopathological Characteristics and Tissue Inhibitor of Metalloproteinase-3 Polymorphism Rs9619311 on Biochemical Recurrence in Taiwanese Patients with Prostate Cancer

**DOI:** 10.3390/ijerph20010306

**Published:** 2022-12-25

**Authors:** Chun-Yu Hsieh, Chia-Yen Lin, Shian-Shiang Wang, Ying-Erh Chou, Ming-Hsien Chien, Yu-Ching Wen, Ming-Ju Hsieh, Shun-Fa Yang

**Affiliations:** 1 Institute of Medicine, Chung Shan Medical University, Taichung 402, Taiwan; 2 Division of Urology, Department of Surgery, Taichung Veterans General Hospital, Taichung 407, Taiwan; 3 Department of Applied Chemistry, National Chi Nan University, Nantou 545, Taiwan; 4 School of Medicine, Chung Shan Medical University, Taichung 402, Taiwan; 5 Graduate Institute of Clinical Medicine, College of Medicine, Taipei Medical University, Taipei 110, Taiwan; 6 Pulmonary Research Center, Wan Fang Hospital, Taipei Medical University, Taipei 116, Taiwan; 7 Department of Urology, Wan Fang Hospital, Taipei Medical University, Taipei 110, Taiwan; 8 Department of Urology, School of Medicine, College of Medicine, Taipei Medical University, Taipei 110, Taiwan; 9 Oral Cancer Research Center, Changhua Christian Hospital, Changhua 500, Taiwan; 10 Department of Post-Baccalaureate Medicine, College of Medicine, National Chung Hsing University, Taichung 402, Taiwan; 11 Graduate Institute of Biomedical Sciences, China Medical University, Taichung 404, Taiwan; 12 Department of Medical Research, Chung Shan Medical University Hospital, Taichung 402, Taiwan

**Keywords:** prostate cancer, TIMP3, polymorphism, biochemical recurrence

## Abstract

The tissue inhibitors of metalloproteinases-3 (TIMP3) are not only endogenous regulators of matrix metalloproteinases (MMPs), but also induce apoptosis and inhibit endothelial cell migration and angiogenesis. The focus of this study was to investigate the relationship between TIMP3 genetic polymorphisms and biochemical recurrence and clinicopathological features of prostate cancer. The TIMP3 rs9619311, rs9862, and rs11547635 genetic polymorphisms were analyzed by real-time polymerase chain reaction to determine their genotypic distributions in 579 patients with prostate cancer. This study found that individuals with the TIMP3 rs9619311 TC or TC + CC genotypes have a significantly higher risk of biochemical recurrence of prostate cancer (*p* = 0.036 and 0.033, respectively). Moreover, in the multivariate analysis, our results showed that pathologic Gleason grade, pathologic T stage, seminal vesicle invasion, lymphovascular invasion, and TIMP3 rs9619311 were associated with increased odds of biochemical recurrence. Patients with a PSA concentration under 7 ng/mL that were found to have the TIMP3 rs9619311 genetic polymorphism were associated with Gleason total score upgrade (*p* = 0.012) and grade group upgrade (*p* = 0.023). Compared with the CC homozygous, the TIMP3 rs9862 CT + TT polymorphic variant was found to be associated with clinically advanced tumor stage (*p* = 0.030) and Gleason total score upgrade (*p* = 0.002) in prostate cancer patients. In conclusion, the results of our study demonstrated that the TIMP3 rs9619311 genetic polymorphism was significantly associated with susceptibility to biochemical recurrence of prostate cancer. TIMP3 genetic polymorphisms, especially rs9619311, can serve as key predictors of biochemical recurrence and disease prognosis of prostate cancer.

## 1. Introduction

Among men worldwide in 2020, prostate cancer is the second most frequent cancer, estimated to have almost 1.4 million new cases [1]. The incidence and mortality from prostate cancer are positively correlated with increasing age, with nearly 60% of incidences occurring in men over the age of 65 [2]. The prevalence of prostate cancer varies widely among different regions and racial groups. Although the incidence and prevalence in Asian men are lower than globally, an upward trend has been observed in most Asian countries over the past few decades [3]. In addition to advanced age and race, the currently well-recognized risk factors for prostate cancer include genetic factors and family history. Other factors that are positively associated with prostate cancer include diet, obesity, smoking, inflammation (chronic inflammation and prostatitis), diabetes, infection, etc. [2]. For the treatment of prostate cancer, significant advances in curative treatment techniques in the last decade, including radical prostatectomy or primary definitive radiotherapy, have improved the efficacy of its treatment, but despite this, biochemical recurrence still occurs in 27–53% of patients [4]. Furthermore, in contrast to other common cancers, the etiology and progression of prostate cancer remains largely unknown.

The tissue inhibitor of metalloproteinases (TIMPs), including TIMP1, 2, 3, and 4, are endogenous regulators of matrix metalloproteinases (MMPs) [5,6,7,8]. TIMPs have been implicated in extracellular matrix degradation, tissue remodeling, cancer cell invasion, and metastasis, and an imbalance between TIMPs and MMPs activity may have implications for cancer progression [9,10,11]. TIMP3 is unique among the mammalian TIMPs, differing from other TIMPs in that it is tightly bound to the extracellular matrix and it has a broader inhibitory activity against MMPs and inhibits it’s closely related a disintegrin and metalloproteinases [12]. In addition, TIMP3 has other biological cellular functions that are unrelated to the inhibition of MMPs, such as the inhibition of endothelial cell migration and the induction of apoptosis [13,14]. There is numerous evidence that has shown that TIMP3 expression is associated with the development and prognosis of multiple human cancers, such as non-small cell lung cancer, colorectal cancer, hepatocellular carcinoma, uterine cervical cancer, and head and neck squamous cell carcinomas, etc. [15,16,17,18,19,20,21,22]. Similarly, the literature studies that are related to prostate cancer confirmed that TIMP3 is a tumor suppressor gene that is frequently downregulated in prostate cancer, and its overexpression can inhibit tumor cell growth and induce apoptosis. TIMP3 expression is also negatively correlated with Gleason score [23,24,25].

Genetic polymorphisms may affect gene expression, mRNA stability, and translational efficiency, and thus may contribute to individual susceptibility to many common diseases, drug metabolism, and genome evolution [26,27]. Our previous study showed that the rs9619311 polymorphism in the promoter region of *TIMP3* was associated with uterine cervical cancer survival and hepatocellular carcinoma susceptibility among women [28,29]. Moreover, according to the data of the international HapMap project and the literature research of Krex et al., two polymorphisms (rs9862 and rs11547635) in the coding exon region of *TIMP3* were identified [30,31]. The rs9862 polymorphism has been reported to play a role in the risk of oral squamous cell carcinoma, lung adenocarcinoma, and uterine cervical cancer, and is associated with prognosis in adenocarcinoma of the gastroesophageal junction [22,28,32,33]. The effect of *TIMP3* genetic polymorphisms on the susceptibility of prostate cancer in the North Indian population has been studied, and the *TIMP3* (1298) C/T polymorphism is known to be significantly associated with the risk of prostate cancer [34]. The roles of *TIMP3* genetic polymorphisms in prostate cancer prognosis, however, have not been investigated. Therefore, three *TIMP3* genetic polymorphisms (rs9619311, rs9862, and rs1154635) were analyzed in this study to elucidate their association with clinicopathological characteristics and biochemical recurrence of prostate cancer in a Taiwanese population.

## 2. Materials and Methods

### 2.1. Subject Selection

From 2012 to 2017, we recruited 579 prostate cancer patients who had undergone robotic-assisted laparoscopic radical prostatectomy and follow up at least 3 years from Taichung Veterans General Hospital in Taiwan. Information was obtained from the medical records of these patients, including age at diagnosis, initial PSA level at diagnosis, clinical and pathologic TNM staging, Gleason grade group, Gleason score, D’Amico classification, and other pathologic features [35]. According to the eighth edition of the American Joint Committee on Cancer staging manual, the TNM staging of patients with prostate cancer was judged. The definition of biochemical recurrence was two consecutive PSA values of >0.2 ng/mL. The definition of the total score upgrade and grade group upgrade were increasing from one prognostic score or grade group to another. This study was approved by the Institutional Review Board of Taichung Veterans General Hospital (IRB No. CE19062A).

### 2.2. Blood Collection and Genomic DNA Extraction

Peripheral blood specimens that were collected from prostate cancer patients were placed in tubes containing ethylenediaminetetraacetic acid, centrifuged, and stored at −80 °C until further analysis. In accordance with the manufacturer’s instructions, genomic DNA from the buffy coat of whole blood specimens was extracted using the QIAamp DNA Blood Mini Kits (Qiagen, Valencia, CA, USA). The final eluted DNA was eluted in TE (Tris-EDTA) buffer and stored at −20 °C before real-time polymerase chain reaction analysis.

### 2.3. Selection and Genotyping Determination of TIMP3 Genetic Polymorphisms

According to the data of the international HapMap project and previous studies, three *TIMP3* genetic polymorphisms were selected in this study, including rs9619311 (promoter region; −1296 T/C), rs9862 (coding exon 3 region; 249 C/T), and rs11547635 (coding exon 3 region; 261 C/T) [20,28,32]. *TIMP3* rs9619311 T allele is a reference allele in the Asian population. *TIMP3* rs9862 and rs11547635 C allele is a reference allele in the Asian population. Assessment of allelic discrimination for the *TIMP3* rs9619311 (assay ID:C_1840822_10), rs9862 (assay ID: C_3294861_10), and rs11547635 (assay ID: C_3294860_10) polymorphisms were performed with the ABI StepOnePlus™ Real-Time PCR System, and ABI TaqMan^®^ SNP Genotyping Assay (Applied Biosystems; Foster City, CA, USA) was used for genotyping according to the manufacturer’s protocols. The final data were collected and further analyzed using ABI StepOnePlus™ software v2.3.

### 2.4. Statistical Analysis

The Student’s *t*-test and chi-square test were used to determine the differences in the distributions of demographical characteristics with or without biochemical recurrence in prostate cancer patients. In the multivariate analysis, logistic regression was used to determine the association of key clinical variables, *TIMP3* genetic polymorphism, and biochemical recurrence. Odds ratios (ORs) and their 95% confidence intervals (CIs) estimated the association between genotypic frequencies, biochemical recurrence, and different clinical status in prostate cancer patients. The SAS statistical software for Windows (version 9.1; SAS Institute, Cary, NC, USA) was used to perform statistical analyses of all data where *p* < 0.05 was defined to have statistically significant difference.

## 3. Results

### 3.1. Clinical Manifestation between the Biochemical Recurrence and without Biochemical Recurrence Group

The distributions of demographical characteristics of patients with prostate cancer are shown in Table 1. Of these, 175 were confirmed to have biochemical recurrence out of 579 prostate cancer patients. Significant differences were observed between patients with or without biochemical recurrence (*p* < 0.001), except for age at diagnosis, total score upgrade, and grade group upgrade. 

### 3.2. The Distribution Frequencies of TIMP3 SNP in Prostate Cancer 

The distribution frequencies of TIMP3 genotypes in prostate cancer patients are presented in Table 2. The genotypic distributions of the TIMP3 SNPs rs9619311, rs9862, and rs11547635 were all consistent with Hardy–Weinberg equilibrium in prostate cancer patients. The highest distribution frequencies of the TIMP3 rs9619311, rs9862, and rs11547635 polymorphisms were the homozygous TT, heterozygous CT, and homozygous CC genotypes, respectively. After adjustment for potential confounders, prostate cancer patients with the TIMP3 rs9619311 polymorphism homozygous for TC and TC + CC had a 1.730-fold (95% CI: 1.035–2.892; *p* = 0.036) and 1.743-fold (95% CI: 1.044–2.910; *p* = 0.033) higher risk of biochemical recurrence compared with those with TT homozygous. However, there were no significant differences between biochemical recurrence group and without biochemical recurrence group in prostate cancer patients with rs9862 and rs11547635 polymorphisms. Moreover, in the multivariate analysis for biochemical recurrence, our results showed that pathologic Gleason grade [OR (95% CI): 1.418 (1.124–1.787); *p* = 0.003], pathologic T stage [OR (95% CI): 1.253 (1.045–1.501); *p* = 0.015], seminal vesicle invasion [OR (95% CI): 2.462 (1.388–4.366); *p* = 0.002], lymphovascular invasion [OR (95% CI): 2.182 (1.181–4.029); *p* = 0.013], and *TIMP3* rs9619311 gene polymorphism [OR (95% CI): 1.337 (1.016–1.760); *p* = 0.038] were associated with increased odds of biochemical recurrence (Table 3). After backward elimination of insignificant clinical variables, pathologic Gleason grade [OR (95% CI): 1.209 (1.056–1.383); *p* = 0.006], pathologic T stage [OR (95% CI): 1.411 (1.195–1.665); *p <* 0.001], seminal vesicle invasion [OR (95% CI): 2.709 (1.556–4.716); *p <* 0.001], lymphovascular invasion [OR (95% CI): 3.005 (1.714–5.267); *p <* 0.001], and *TIMP3* rs9619311 gene polymorphism [OR (95% CI): 1.348 (1.030–1.764); *p* = 0.029] were also associated with increased odds of biochemical recurrence (Table 3).

### 3.3. The Role of TIMP3 Genetic Polymorphisms in the Clinical Status of Prostate Cancer

To clarify the role of TIMP3 genetic polymorphisms in the clinical status of prostate cancer, the distribution frequencies of the clinical status and TIMP3 genotypic frequencies were estimated in 579 patients with prostate cancer. However, the TIMP3 rs9619311 gene polymorphisms showed no significant association with the clinical status of prostate cancer patients (Table 4). Furthermore, as shown in Table 5, we observed a significant association between the TIMP3 rs9862 gene polymorphism and clinical status (clinical T stage and total score upgrade) in prostate cancer patients (*p* = 0.030 and 0.002, respectively).

We further analyzed the distribution of the clinical status and TIMP3 rs9619311, rs9862, and rs1154635 genotypic frequencies in 153 patients with prostate cancer with PSA concentrations under 7 ng/mL. Interestingly, we found that an analysis of the association between the TIMP3 rs9619311 polymorphism and patients with PSA concentrations under 7 ng/mL revealed significant differences in the total score upgrade and grade group upgrade (*p* = 0.012 and 0.023, respectively; Table 6).

## 4. Discussion

In this study, we explored the association of *TIMP3* genetic polymorphisms with biochemical recurrence and clinicopathological characteristics in patients with prostate cancer. The age at diagnosis and incidence of biochemical recurrence in the cohort that we recruited were consistent with previous reports, with nearly 60% occurring in men over 65 years of age and a biochemical recurrence rate of approximately 30.2% (175/579) [2,4]. In this analysis, we observed no statistically significant differences in age at diagnosis, Gleason total score upgrade, and grade group upgrade among prostate cancer patients with or without biochemical recurrence (*p* = 0.589, 0.150, 0.540; Table 1), which indicated that these are associated with progression but not recurrence of prostate cancer.

A disruption of the balance between MMPs and TIMPs has been implicated in the progression of multiple cancers [36,37,38]. Among them, TIMP3, the most common TIMP that is found in different types of cancer, is considered a marker of good prognosis because it prevents disease progression [37]. However, the roles of *TIMP3* genetic polymorphisms in prostate cancer prognosis have not been investigated. We further evaluated the association of the genotype distributions of *TIMP3* genetic polymorphisms with the biochemical recurrence of prostate cancer in a Taiwanese population-based study. The rs9619311 polymorphism in the promoter region of *TIMP3* and other two polymorphisms (rs9862 and rs11547635) have been reported to play a role in the risk of various cancers [22,28,29,32,33]. Of the three *TIMP3* genetic polymorphisms that were selected in this study, only the rs9619311 polymorphism was observed to be significantly associated with the biochemical recurrence of prostate cancer (Table 2). In a literature review by Shinojima et al., the results indicate that histone modifications can contribute to TIMP3 repression in the absence of promoter hypermethylation in prostate cancer [25]. Similarly, the literature studies that are related to oral cancer confirmed that the suppression of TIMP3 by DNA methylation contributes to cancer metastasis [19] and oral cancer patient who carry *TIMP3* rs9862 polymorphism CT and TT polymorphic variants have significantly higher plasma levels of TIMP3 expression [20]. Therefore, the association between *TIMP3* genetic variants and TIMP3 gene expression on the risk of biochemical recurrence of prostate cancer warrant further investigation.

Previous literature studies have shown that the C allele of *TIMP3* rs9619311 is associated with poor five-year survival in uterine cervical cancer patients, increased risk of breast cancer, and protection against hepatocellular carcinoma in women [28,29,39]. Although the *TIMP3* rs9619311 polymorphism was not significantly associated with the clinicopathological characteristics of the 579 prostate cancer patients in this study (Table 4), it was associated with total score upgrade (*p* = 0.012; OR: 3.007; 95% CI: 1.243–7.274) and grade group upgrade (*p* = 0.023; OR: 2.724; 95% CI: 1.128–6.581) in cohort with PSA concentrations under 7 ng/mL (Table 6). From this, it can be seen that the *TIMP3* rs9619311 also plays an important role in prostate cancer, and patients with the “TC + CC” polymorphic variant are at high risk for both cancer progression and biochemical recurrence.

Multiple studies have reported that the T allele of *TIMP3* rs9862 is associated with a higher risk of developing cancer and decreased survival [20,28,32]. Among them, the CT/TT genetic polymorphism has poor cellular differentiation of cervical cancer, and a higher risk of developing EGFR mutation, advanced stage, and lymph node metastasis in lung adenocarcinoma [28,32]. In a literature review by Su et al., there was a significant association between harboring TT homozygotes of rs9862 and developing large tumors in patients with oral cancer [20]. In this study, there were the same analysis results, which also confirmed that the rs9862 CT + TT allele carriers developed a higher proportion of advanced tumors (clinical T stage 3 + 4) (*p* = 0.030; OR: 1.886; 95% CI: 1.055–3.369) in prostate cancer (Table 5). However, it is worth noting that prostate cancer patients in the CT + TT genotypic polymorphism group were less likely to have an upgrade of the total score (*p* = 0.002; OR: 0.571; 95% CI: 0.399–0.819). However, cancers with higher Gleason scores are known to be more aggressive and have worse prognosis. Whether such results are related to the fact that the rs9862 polymorphism, although important in cancer progression, is not significantly associated with biochemical recurrence, needs to be further explored. 

Prostate cancer has previously been studied and it has been found that individuals with the *TIMP3* (1298) C/T polymorphism have a reduced risk of prostate cancer, but no association with tumor development has been found [34]. Due to the small sample size and the study of only one *TIMP3* genetic polymorphism, the association between *TIMP3* and prostate cancer cannot be fully presented. Therefore, we investigated increasing the sample size and the three *TIMP3* genetic polymorphisms that are known to be strongly associated with cancer for further analysis. However, our study is still limited by the lack of tumor specimens and information on *TIMP3* expression levels from patients with prostate cancer. In addition, further in-depth analysis of the effects of different *TIMP3* genetic polymorphisms and their mRNA and protein expression levels on tumor progression, biochemical recurrence, and disease prognosis of prostate cancer is required.

## 5. Conclusions

In conclusion, our results suggest that *TIMP3* rs9619311 genetic polymorphisms are associated with the risk of biochemical recurrence in patients with prostate cancer. Although other *TIMP3* genetic polymorphisms may have limited direct effects on biochemical recurrence of prostate cancer, both *TIMP3* rs9619311 and rs9862 genetic polymorphisms can be used as important predictors of prostate cancer prognosis, and among them, the rs9619311 gene polymorphism can also be used as a pivotal predictor of biochemical recurrence of prostate cancer.

## Figures and Tables

**Table 1:** The distributions of demographical characteristics in 579 patients with prostate cancer.

Variable	Biochemical Recurrence		*p*-Value
	No (*n* = 404)	Yes (*n* = 175)	
**Age at diagnosis (years)**			
≤65	168 (41.6%)	77 (44.0%)	*p* = 0.589
>65	236 (58.4%)	98 (56.0%)	
**PSA at diagnosis (ng/mL)**			
≤7	134 (33.2%)	19 (10.9%)	*p* < 0.001 *
7–10	84 (20.8%)	33 (18.9%)	
>10	186 (46.0%)	123 (70.3%)	
**Pathologic Gleason grade group**			
1 + 2 + 3	367 (90.8%)	117 (66.9%)	*p* < 0.001 *
4 + 5	37 (9.2%)	58 (33.1%)	
**Clinical T stage**			
1 + 2	369 (91.3%)	132 (75.4%)	*p* < 0.001 *
3 + 4	35 (8.7%)	43 (24.6%)	
**Pathologic T stage**			
2	266 (65.8/%)	40 (22.9%)	*p* < 0.001 *
3 + 4	138 (34.2%)	135 (77.1%)	
**Pathologic N stage**			
N0	393 (97.3%)	137 (78.3%)	*p* < 0.001 *
N1	11 (2.7%)	38 (21.7%)	
**Seminal vesicle invasion**			
No	364 (90.1%)	88 (50.3%)	*p* < 0.001 *
Yes	40 (9.9%)	87 (49.7%)	
**Perineural invasion**			
No	140 (34.7%)	15 (8.6%)	*p* < 0.001 *
Yes	265 (65.3%)	160 (91.4%)	
**Lymphovascular invasion**			
No	373 (92.3%)	109 (62.3%)	*p* < 0.001 *
Yes	31 (7.7%)	66 (37.7%)	
**D’Amico classification**			
Low risk	55 (13.6%)	5 (2.9%)	*p* < 0.001 *
Intermediate risk	168 (41.6%)	52 (29.7%)	
High risk	181 (44.8%)	118 (67.4%)	
**Total score upgrade**			
No	247 (61.1%)	118 (67.4%)	*p* = 0.150
Yes	157 (38.9%)	57 (32.6%)	
**Grade group upgrade**			
No	236 (58.4%)	107 (61.1%)	*p* = 0.540
Yes	168 (41.6%)	68 (38.9%)	

The adjusted odds ratios (AORs) with their 95% confidence intervals (CIs) were estimated by multiple logistic regression models after controlling for age at diagnosis, PSA levels at diagnosis, pathologic Gleason grade group, clinical T stage, pathologic T stage, pathologic N stage, seminal vesicle invasion, perineural invasion, lymphovascular invasion, D’Amico classification, total score upgrade, and grade group upgrade. * *p*-value < 0.05 as statistically significant.

**Table 2:** Distribution frequency of *TIMP3* genotypes in 579 patients with prostate cancer.

Variable	Biochemical Recurrence		AOR (95% CI)	*p*-Value
	No (*n* = 404)	Yes (*n* = 175)		
**rs9619311**				
TT	340 (84.2%)	134 (76.6%)	1.00	
TC	64 (15.8%)	40 (22.9%)	1.730 (1.035–2.892)	0.036 *
CC	0 (0.0%)	1 (0.5%)	---	---
TC + CC	64 (15.8%)	41 (23.4%)	1.743 (1.044–2.910)	0.033 *
**rs9862**				
CC	132 (32.7%)	48 (27.4%)	1.00	
CT	200 (49.5%)	96 (54.9%)	1.435 (0.885–2.326)	0.143
TT	72 (17.8%)	31 (17.7%)	1.266 (0.674–2.377)	0.463
CT + TT	272 (67.3%)	127 (72.6%)	1.390 (0.877–2.204)	0.161
**rs11547635**				
CC	186 (46.0%)	87 (49.7%)	1.00	
CT	171 (42.3%)	66 (37.7%)	0.764 (0.491–1.189)	0.234
TT	47 (11.7%)	22 (12.6%)	0.952 (0.489–1.856)	0.886
CT + TT	218 (54.0%)	88 (50.3%)	0.803 (0.531–1.214)	0.298

* *p*-value < 0.05 as statistically significant.

**Table 3:** The multivariate analysis for biochemical recurrence in 579 patients with prostate cancer.

Variable	OR (95% CI)	*p*-Value	OR (95% CI) ^a^	*p*-Value ^a^
PSA at diagnosis	1.391 (0.857–2.256)	0.181		
Pathologic Gleason grade group	1.418 (1.124–1.787)	0.003 *	1.209 (1.056–1.383)	0.006 *
Clinical T stage	1.073 (0.863–1.334)	0.525		
Pathologic T stage	1.253 (1.045–1.501)	0.015 *	1.411 (1.195–1.665)	<0.001 *
Pathologic N stage	2.099 (0.876–5.029)	0.096		
Seminal vesicle invasion	2.462 (1.388–4.366)	0.002 *	2.709 (1.556–4.716)	<0.001 *
Perineural invasion	1.860 (0.972–3.562)	0.061		
Lymphovascular invasion	2.182 (1.181–4.029)	0.013 *	3.005 (1.714–5.267)	<0.001 *
D’Amico classification	0.918 (0.710–1.188)	0.516		
rs9619311 (TT vs. TC + CC)	1.337 (1.016–1.760)	0.038 *	1.348 (1.030–1.764)	0.029 *
rs9862 (CC vs. CT + TT)	1.199 (0.931–1.544)	0.160	1.212 (0.942–1.559)	0.135
rs11547635 (CC vs. CT + TT)	1.016 (0.802–1.287)	0.897	1.049 (0.830–1.326)	0.688

^a^ after backward elimination of insignificant clinical variables. The ORs with the analyzed 95% CIs were estimated by logistic regression models. * *p*-value < 0.05 as statistically significant.

**Table 4:** Odds ratios (OR) and 95% confidence intervals (CI) of the clinical status and *TIMP3* rs9619311 genotypic frequencies in 579 patients with prostate cancer.

Variable	Genotypic Frequencies			
rs9619311	TT (*n* = 474)	TC + CC (*n* = 105)	OR (95% CI)	*p*-Value
**Pathologic Gleason grade group**				
1 + 2 + 3	400 (84.4%)	84 (80.0%)	1.00	*p* = 0.272
4 + 5	74 (15.6%)	21 (20.0%)	1.351 (0.789–2.316)	
**Clinical T stage**				
1 + 2	409 (86.3%)	92 (87.6%)	1.00	*p* = 0.718
3 + 4	65 (13.7%)	13 (12.4%)	0.889 (0.470–1.681)	
**Pathologic T stage**				
2	255 (53.8%)	51 (48.6%)	1.00	*p* = 0.332
3 + 4	219 (46.2%)	54 (51.4%)	1.233 (0.808–1.882)	
**Pathologic N stage**				
N0	437 (92.2%)	93 (88.6%)	1.00	*p* = 0.228
N1	37 (7.8%)	12 (11.4%)	1.524 (0.766–3.034)	
**Seminal vesicle invasion**				
No	375 (79.1%)	77 (73.3%)	1.00	*p* = 0.195
Yes	99 (20.9%)	28 (26.7%)	1.377 (0.847–2.239)	
**Perineural invasion**				
No	132 (27.8%)	23 (21.9%)	1.00	*p* = 0.213
Yes	342 (72.2%)	82 (78.1%)	1.376 (0.831–2.278)	
**Lymphovascular invasion**				
No	392 (82.7%)	90 (85.7%)	1.00	*p* = 0.454
Yes	82 (17.3%)	15 (14.3%)	0.797 (0.439–1.446)	
**D’Amico classification**				
Low/Intermediate risk	229 (48.3%)	51 (48.6%)	1.00	*p* = 0.962
High risk	245 (51.7%)	54 (51.4%)	0.990 (0.648–1.511)	
**Total score upgrade**				
No	304 (64.1%)	61 (58.1%)	1.00	*p* = 0.246
Yes	170 (35.9%)	44 (41.9%)	1.290 (0.838–1.984)	
**Grade group upgrade**				
No	284 (59.9%)	59 (56.2%)	1.00	*p* = 0.482
Yes	190 (40.1%)	46 (43.8%)	1.165 (0.760–1.786)	

The ORs with the analyzed 95% CIs were estimated by logistic regression models.

**Table 5:** Odds ratios (OR) and 95% confidence intervals (CI) of the clinical status and *TIMP3* rs9862 genotypic frequencies in 579 patients with prostate cancer.

Variable	Genotypic Frequencies			
rs9862	CC (*n* = 180)	CT + TT (*n* = 399)	OR (95% CI)	*p*-Value
**Pathologic Gleason grade group**				
1 + 2 + 3	149 (82.8%)	335 (84.0%)	1.00	*p* = 0.722
4 + 5	31 (17.2%)	64 (16.0%)	0.918 (0.574–1.470)	
**Clinical T stage**				
1 + 2	164 (91.1%)	337 (84.5%)	1.00	*p* = 0.030 *
3 + 4	16 (8.9%)	62 (15.5%)	1.886 (1.055–3.369)	
**Pathologic T stage**				
2	95 (52.8%)	211 (52.9%)	1.00	*p* = 0.981
3 + 4	85 (47.2%)	188 (47.1%)	0.996 (0.700–1.417)	
**Pathologic N stage**				
N0	168 (93.3%)	362 (90.7%)	1.00	*p* = 0.297
N1	12 (6.7%)	37 (9.3%)	1.431 (0.728–2.814)	
**Seminal vesicle invasion**				
No	143 (79.4%)	309 (77.4%)	1.00	*p* = 0.590
Yes	37 (20.6%)	90 (22.6%)	1.126 (0.732–1.732)	
**Perineural invasion**				
No	41 (22.8%)	114 (28.6%)	1.00	*p* = 0.145
Yes	139 (77.2%)	285 (71.4%)	0.737 (0.489–1.112)	
**Lymphovascular invasion**				
No	151 (83.9%)	331 (83.0%)	1.00	*p* = 0.781
Yes	29 (16.1%)	68 (17.0%)	1.070 (0.665–1.721)	
**D’Amico classification**				
Low/Intermediate risk	87 (48.3%)	193 (48.4%)	1.00	*p* = 0.993
High risk	93 (51.7%)	206 (51.6%)	0.998 (0.702–1.420)	
**Total score upgrade**				
No	97 (53.9%)	268 (67.2%)	1.00	*p* = 0.002 *
Yes	83 (46.1%)	131 (32.8%)	0.571 (0.399–0.819)	
**Grade group upgrade**				
No	96 (53.3%)	247 (61.9%)	1.00	*p* = 0.052
Yes	84 (46.7%)	152 (38.1%)	0.703 (0.493–1.004)	

The ORs with the analyzed 95% CIs were estimated by logistic regression models. * *p*-value < 0.05 as statistically significant.

**Table 6:** Odds ratios (OR) and 95% confidence intervals (CI) of the clinical status and *TIMP3* rs9619311 genotypic frequencies in 153 patients with prostate cancer with a PSA concentration under 7 ng/mL.

Variable	Genotypic Frequencies			
rs9619311	TT (*n* = 127)	TC + CC (*n* = 26)	OR (95% CI)	*p*-Value
**Pathologic Gleason grade group**				
1 + 2 + 3	118 (92.9%)	24 (92.3%)	1.00	*p* = 0.913
4 + 5	9 (7.1%)	2 (7.7%)	1.093 (0.222–5.378)	
**Clinical T stage**				
1 + 2	119 (93.7%)	25 (96.2%)	1.00	*p* = 0.628
3 + 4	8 (6.3%)	1 (3.8%)	0.595 (0.071–4.972)	
**Pathologic T stage**				
2	98 (77.2%)	16 (61.5%)	1.00	*p* = 0.096
3 + 4	29 (22.8%)	10 (38.5%)	2.112 (0.865–5.154)	
**Pathologic N stage**				
N0	121 (95.3%)	25 (96.2%)	1.00	*p* = 0.845
N1	6 (4.7%)	1 (3.8%)	0.807 (0.093–6.997)	
**Seminal vesicle invasion**				
No	121 (95.3%)	24 (92.3%)	1.00	*p* = 0.536
Yes	6 (4.7%)	2 (7.7%)	1.681 (0.320–8.831)	
**Perineural invasion**				
No	45 (35.4%)	8 (30.8%)	1.00	*p* = 0.649
Yes	82 (64.6%)	18 (69.2%)	1.235 (0.498–3.064)	
**Lymphovascular invasion**				
No	119 (93.7%)	26 (100.0%)	---	---
Yes	8 (6.3%)	0 (0.0%)	---	
**D’Amico classification**				
Low/Intermediate risk	89 (70.1%)	16 (61.5%)	1.00	*p* = 0.393
High risk	38 (29.9%)	10 (38.5%)	1.464 (0.609–3.517)	
**Total score upgrade**				
No	78 (61.4%)	9 (34.6%)	1.00	*p* = 0.012 *
Yes	49 (38.6%)	17 (65.4%)	3.007 (1.243–7.274)	
**Grade group upgrade**				
No	75 (59.1%)	9 (34.6%)	1.00	*p* = 0.023 *
Yes	52 (40.9%)	17 (65.4%)	2.724 (1.128–6.581)	

The ORs with the analyzed 95% CIs were estimated by logistic regression models. * *p*-value < 0.05 as statistically significant.

## Data Availability

The datasets that were generated for this study are available on request to the corresponding authors.
